# Endometrial Receptivity Analysis (ERA): data versus opinions

**DOI:** 10.1093/hropen/hoab011

**Published:** 2021-04-14

**Authors:** Maria Ruiz-Alonso, Diana Valbuena, Carlos Gomez, Juliana Cuzzi, Carlos Simon

**Affiliations:** 1 Igenomix Foundation-INCLIVA, Valencia, Spain; 2 Igenomix SL, Valencia, Spain; 3 Igenomix USA & Canada, Miami, FL, USA; 4 Department of Pediatrics, Obstetrics & Gynecology, University of Valencia, Valencia, Spain; 5 Department of Obstetrics and Gynecology, BIMDC, Harvard University, Boston, MA, USA

**Keywords:** endometrial receptivity, embryo transfer, endometrium, implantation / recurrent implantation failure

## Abstract

This article summarises and contextualises the accumulated basic and clinical data on the ERA test and addresses specific comments and opinions presented by the opponent as part of an invited debate. Progress in medicine depends on new technologies and concepts that translate to practice to solve long-standing problems. In a key example, combining RNA sequencing data (transcriptomics) with artificial intelligence (AI) led to a clinical revolution in personalising disease diagnosis and fostered the concept of precision medicine. The reproductive field is no exception. Translation of endometrial transcriptomics to the clinic yielded an objective definition of the limited time period during which the maternal endometrium is receptive to an embryo, known as the window of implantation (WOI). The WOI is induced by the presence of exogenous and/or endogenous progesterone (P) after proper oestradiol (E_2_) priming. The window lasts 30–36 hours and, depending on the patient, occurs between LH + 6 and LH + 9 in natural cycles or between P + 4 and P + 7 in hormonal replacement therapy (HRT) cycles. In approximately 30% of IVF cycles in which embryo transfer is performed blindly, the WOI is displaced and embryo-endometrial synchrony is not achieved. Extending this application of endometrial transcriptomics, the endometrial receptivity analysis (ERA) test couples next-generation sequencing (NGS) to a computational predictor to identify transcriptomic signatures for each endometrial stage: proliferative (PRO), pre-receptive (PRE), receptive (R) and post-receptive (POST). In this way, personalised embryo transfer (pET) may be possible by synchronising embryo transfer with each patient’s WOI. Data are the only way to confront arguments sustained in opinions and/or misleading concepts; it is up to the reader to make their own conclusions regarding its clinical utility.

## Introduction

Despite its many advances and achievements, reproductive medicine has long neglected the endometrial factor. Indeed, since the inception of this field, the oocyte/embryo has remained the central focus. In contrast, the maternal endometrium was considered a passive part of the reproductive process: a ‘good embryo’ (or four or five) was all that mattered. Yet, while embryology and embryo transfer technologies have improved considerably over the past 30 years, the efficacy of IVF remains low worldwide, with current live birth rates of 25–30% per started cycle (Adamson *et al.*, 2018). At least part of this gap may derive from a failure to consider the endometrium; after all, it is fair to say that any process relying on a collaboration between partners requires the function and coordination of both.

Further progress in reproductive medicine, like in all of medicine, depends on bringing new technologies and concepts to bear on long-standing problems. In recent decades, transcriptomics or RNA sequencing, has emerged as a powerful tool for clinical diagnosis of disease (Byron *et al.*, 2016). Applications of transcriptomics are found in cancer (Ferreira *et al.*, 2014; Tan *et al.*, 2016), cardiovascular pathologies (Matsa *et al.*, 2016) and neurodegenerative diseases (Ferreiro *et al.*, 2012), among others. The reproductive medicine field is no exception.

The endometrial receptivity analysis (ERA) was first published ten years ago (Díaz-Gimeno *et al.*, 2011) after more than ten years of basic and translational research by a handful of pioneers, including our group. The research objective was to consider the endometrial factor and determine the potential to personalise this in the IVF workup, to ultimately synchronise embryo transfer to a receptive maternal endometrium. Since then, personalised medicine for the endometrial factor has taken off, changing the clinical practice of more than 4000 reproductive clinics in more than 90 countries worldwide. Below, we summarise the concepts, data and clinical applications for the ERA.

## A decade of basic research leading to transcriptomic characterisation of the human endometrium

In the 2000s, endometrial dating by histological evaluation (Noyes *et al.*, 1950) was used as a predictor of endometrial receptivity or fertility status (Coutifaris *et al.*, 2004; Murray *et al.*, 2004). This led to an absence of any reliable diagnostic test to determine the endometrial status. Consequently, the standard workup for infertility in clinics worldwide no longer included endometrial status, beyond a limited use of imaging to determine endometrial thickness and pattern. The frequently reported cut-off of 7 mm seems not to be justified to decide on cycle cancellation or to refrain from further IVF, nor to guide embryo transfer (Kasius *et al.*, 2014).

With the arrival of the genomics revolution, endometrial biology became deeply scrutinised. Four independent groups simultaneously reported on transcriptomic profiling of the secretory phase of the human endometrium in natural cycles, searching for the window of implantation (WOI) (Kao *et al.*, 2002; Carson *et al.*, 2002; Riesewijk *et al.*, 2003; Mirkin *et al.*, 2005). Two other groups extended this transcriptomic characterisation across the menstrual cycle (Borthwick *et al.*, 2003; Ponnampalam *et al.*, 2004). Subsequent studies were extended to ovarian stimulation cycles (Mirkin *et al.*, 2004; Horcajadas *et al.*, 2005; Simon *et al.*, 2005), and even refractory cycles in patients with inert intrauterine devices (IUD) (Horcajadas *et al.*, 2006) (for review see Horcajadas *et al.*, 2007). Since 2005, myriad papers have further described the transcriptomic profile across the menstrual cycle (Mirkin *et al.*, 2005; Punyadeera *et al.*, 2005; Simon *et al.*, 2005; Yanaihara *et al.*, 2005; Talbi *et al.*, 2006; Critchley *et al.*, 2006; Horcajadas *et al.*, 2008; Haouzi *et al.*, 2009; Kuokkanen *et al.*, 2010; Tseng *et al.*, 2010; Van Vaerenbergh *et al.*, 2010; Revel *et al.*, 2011). The next step was a comparison of endometrial profiles between fertile patients and those with pathologies such as recurrent implantation failure (Tapia *et al.*, 2008; Koler *et al.*, 2009; Altmäe *et al.*, 2010; Macklon, 2017), endometrial cancer (Habermann *et al.*, 2011), endometriosis (Matsuzaki, 2011; Garcia-Velasco, 2015), and obesity (Comstock *et al.*, 2017). This progress thereby facilitated the transition from anatomical to molecular medicine of the endometrial factor and ultimately paved the way for its clinical application.

## Endometrial receptivity analysis (ERA)

The ERA was the first transcriptomic test developed to diagnose the endometrial receptivity status of infertile patients (Díaz-Gimeno *et al.*, 2011). To identify genes involved in the human endometrial receptivity signature, we initially analysed differences in genome-wide expression profiles between receptive and pre-receptive endometrium using raw expression data from three different models of endometrial receptivity: the natural cycle as the optimal model, the ovarian stimulation cycle as suboptimal, and the refractory endometrium induced by the insertion of an IUD as a negative control (for review see Ruiz-Alonso *et al.*, 2012). We performed a *t*-test and selected genes showing an absolute fold-change >3 and a false discovery rate <0.05. Three different statistical approaches were employed, the union of the T-Rex gene list (GEPAS) (http://gepas.bioinfo.cipf.es/) and the SAM gene list (http://www.stat.stanford.edu/_tibs/SAM/), intersected with the multitest gene list (http://www.bioconductor.org/). Mathematically, the approach can be written as: [T-Rex U SAM]Xmulttest.

Initially, the ERA was created as a customised array containing 238 differentially expressed genes that were coupled to a computational predictor able to identify the transcriptomic profiles of proliferative (PRO), pre-receptive (PRE), receptive (R) or post-receptive (POST) endometrial samples, regardless of their histological appearance. These 238 genes were presented to the scientific community in Díaz-Gimeno *et al.* (2011). But even more important than the genes implicated is the prediction algorithm, which enables combining the expression of all 238 analysed genes to reach a consensus clinical diagnosis.

To test its accuracy and reproducibility, ERA was compared to standard histological methods in endometrial biopsies collected throughout the menstrual cycle (n = 128), and results were measured by the quadratic weighted Kappa index (Diaz-Gimeno *et al.*, 2013). For the accuracy study, biopsies were grouped into two cohorts: the training set (n = 79) for ERA machine-learning training and dating, and a test set (n = 49) for comparison between histological and ERA dating. For the reproducibility study, seven women underwent one ERA test and a repeat test 29–40 months later on the same day of their cycle. Concordance values following luteinising hormone (LH) peak were 0.618 (0.446–0.791) and 0.685 (0.545–0.824) for the two pathologists. Further, the Kappa index for inter-observer variability (0.622; 0.435–0.839) was sub-optimal. ERA dating achieved a concordance of 0.922 (0.815–1.000) with LH peak. ERA test reproducibility in the indicated subgroup was consistent in all patients (Diaz-Gimeno *et al.*, 2013). These data provided robust indicators for the utility of ERA.

## A decade of ERA clinical application

**Figure 1.**
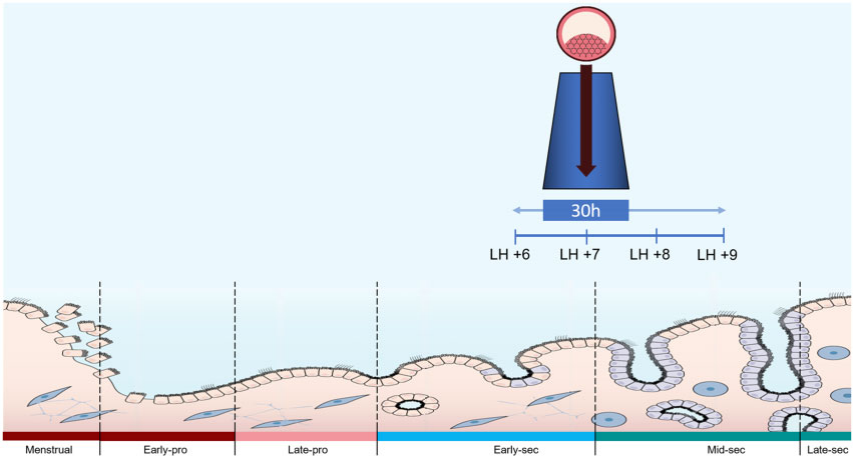
**Diagram representing duration and timing of the window of implantation (WOI).** The WOI lasts approximately 30–36 hours and, depending on the patient, occurs between LH + 6 and LH + 9 in natural cycles or between P + 4 and P + 7 in hormonal replacement therapy (HRT) cycles.

The WOI lasts 30–36 hours and, depending on the patient, occurs between LH + 6 to LH + 9 in natural cycles or from P + 4 to P + 7 in hormonal replacement therapy (HRT) cycles (Rincon *et al.*, 2018) (Fig. 1).

The initial ERA proof of concept in Caucasian patients with recurrent implantation failure (RIF) was published in 2013 (Ruiz-Alonso *et al.*, 2013) in a prospective multicentre interventional clinical trial. Our hypothesis was that implantation failure of endometrial origin is not a pathology or an endometrial dysfunction (conditions that stigmatise a patient), but rather a failure to synchronise the developing embryo with a patient’s individual WOI. The study group included 85 patients with RIF (4.8 ± 2.0 previous failed cycles) and at least four total morphologically high-grade embryos or blastocysts transferred and no other explanation for the implantation failures. The control group was 25 patients. We detected that 25.9% of patients with RIF showed a displaced WOI (advanced or delayed), while only 12% of control patients had such displacement. Therefore, we concluded that one in four patients with RIF have a displaced/asynchronous WOI. Our computational algorithm classified these patients as non-receptive endometrium either pre- (84%) or post-receptive (16%), which was further verified by a second ERA test. We translated these genomic results to the clinic by transferring embryo(s) according to the WOI of the individual patient, providing a ‘personalised embryo transfer’ (pET) resulting in a 50.0% pregnancy rate (PR) and 38.5% implantation rate (IR), similar to that of controls. These results suggested that normal pregnancy and implantation rates may be achieved in patients with RIF of endometrial origin if synchrony between the embryo and receptive endometrium is accomplished (Ruiz-Alonso *et al.*, 2013).

This initial study was further validated by the report of a clinical case of successful pET after seven previous failed IVF attempts (four with autologous oocytes and three with donor oocytes) (Ruiz-Alonso *et al.*, 2014a). The case report was soon complemented by a pilot study of 17 patients undergoing oocyte donation who experienced from 1 to 6 failed implantations (2.9 ± 2.1) with routine embryo transfer (ET), but were subsequently treated with pET after diagnosis of their WOI. Results after pET showed that these patients (with up to six previous failures) reached a 60% clinical PR, while a 19% PR was achieved after routine ET in a non-receptive endometrium diagnosed by ERA (Ruiz-Alonso *et al.*, 2014a).

After these initial reports, independent groups started to publish their own data using ERA to guide pET in their clinical practice. In 2015, a retrospective study in an Indian population (Mahajan, 2015) analysed data from three different groups: patients with RIF, patients with one previous failed cycle, and patients with atrophic endometrium (<6 mm). Their results revealed that 27.5% of patients with RIF had a displaced WOI, while only 15% of patients with one previous failure had a displacement (similar to our data published in 2013). After pET, the overall ongoing PR in the RIF group was 42.4% and IR was 33%, which was similar to that in the group of patients with one failure. This finding again suggested that results in patients with RIF can be normalised after pET. Interestingly, the ERA test revealed displaced WOIs in 25% of those with atrophic endometrium, but after pET their PR was 66.7% despite having an endometrial thickness <6 mm. Similar cases have been reported for unresponsive 4-mm endometrium (Cruz and Bellver, 2014). Intriguingly, in patients with congenital uterine abnormalities such as uterus didelphys and with previous failed ETs, different endometrial receptivity status was found in each hemiuterus (Carranza *et al.*, 2018).

In 2017, a retrospective analysis of 50 patients with RIF assessed the impact of pET guided by ERA in a Japanese population (Hashimoto *et al.*, 2017). Approximately 24% of patients in the RIF group had a displaced WOI, but after pET they reached a 50% PR, similar to that reported in previous studies. In 2019, Hromadova *et al.* (2019) reported similar findings in the Czech Republic. Retrospective data from 85 patients (74 RIF cases and 11 controls) revealed that 36.5% of RIF patients showed a displaced WOI and 69.2% became pregnant after performing pET guided by ERA. Ota *et al.* (2019) published a case report of a Japanese patient who achieved pregnancy with pET guided by ERA after 11 previous failed attempts. Simrandeep and Padmaja (2019) reported three severe cases of RIF in Indian patients; two of the patients had a previous ERA performed at a different centre, and the recommendation for pET for a displaced WOI was not followed, resulting another failure. Once pET was implemented, successful clinical pregnancies were achieved in both patients.

While these studies indicate the outcomes for patients who received pET based on their WOI, what is the clinical outcome in patients in whom transfers occur outside of their WOI according to ERA? Such data were collected in a study comparing the clinical outcome of pET in 205 receptive (R) patients versus embryo transfers performed in 52 non-receptive (NR) patients according to the ERA test. The clinical outcome was 23% PR and 13% IR after transfer in the NR phase, with 0% ongoing pregnancy rate (OPR); in contrast, when pET was performed based on the R phase, 60% PR, 45% IR and 74% OPR were achieved (Ruiz-Alonso *et al.*, 2014b).

However, other retrospective publications have not found statistical clinical differences in pET versus ET in patients with RIF (Patel *et al.*, 2019). Tan *et al.* observed that when embryos were chromosomally analysed, a higher IR and OPR was observed in pET versus ET (66.7 vs. 44.4% and 58.3 vs. 33.3%, respectively), but these differences were not statistically significant due to the small sample size (Tan *et al.*, 2018). Some authors undertook a different approach to evaluate the clinical efficiency of ERA, using retrospective cohort studies comparing patients with an indication of ERA treated by pET to those without an ERA indication, and yielding similar clinical results between these groups (Bassil *et al.*, 2018; Neves *et al.*, 2019; Cozzolino *et al.*, 2020). We should bear in mind that, until 2020, patients with indication for ERA were the most difficult cases with several previous failures, as no explanation was found for their RIF of endometrial origin even after a through infertility workup. Therefore, the fact that pET in this RIF population was able to obtain similar clinical results to those in ‘control patients’ is confirmatory of previous results, due to improved outcomes for the most difficult patients.

Recently, we explored the effectiveness of personalized embryo transfer guided by ERA compared to frozen ET (FET) or fresh embryo transfer (ET) (Simón *et al.*, 2020). This prospective open label randomised clinical trial (RCT) included 458 patients younger than 37 years undergoing IVF with blastocyst transfer at their first appointment, across 16 reproductive centres from Europe, America and Asia, and involved 30 co-authors together with the support of the ERA RCT Consortium. Intention-to-treat analysis revealed comparable clinical outcomes across transfer types; however, there was a significantly higher cumulative pregnancy rate (CPR) in the pET group (93.6%) than in FET (79.7%) (*P* = 0.0005) and ET (80.7%) groups (*P* = 0.0013). By per-protocol analysis, pET resulted in a 56.2% live-birth (LB) rate after first embryo transfer compared to 42.4% for FET (*P* = 0.09) and 45.7% for ET (45.7%, *P* = 0.17). After 12 months, pET resulted in significantly higher cumulative LB rate (71.2%) compared to FET (55.4%, *P* = 0.04) and ET (48.9%, *P* = 0.003). pET also yielded significantly higher PR at the first embryo transfer (72.5%) compared to FET (54.3%, *P* = 0.01) and ET (58.5%, *P* = 0.05). Similar outcomes were observed for first-transfer IRs, which were 57.3% for pET versus 43.2% (*P* = 0.03) and 38.6% (*P* = 0.004) for FET and ET, respectively. All groups exhibited similar obstetrical, delivery type and neonatal outcomes. While the RCT experienced an unexpectedly high patient drop-out (observed, 50%; expected, 30%), the per-protocol analysis comparing pET to FET and ET arms revealed significantly better cumulative LB rates, PR and IR. These findings support that using the ERA test at the first appointment to guide pET may have clinical benefit. Further, an independent RCT comparing frozen blastocyst transfer using conventional timing versus timing guided by ERA is under way (ClinicalTrials.gov Identifier: NCT03558399).

## Number of genes tested

Discovery of the genes involved in endometrial receptivity has been challenging. The background presented above further encompasses that the sets of genes identified within different transcriptomic studies differs due to differences in experimental designs, type of array initially used, sampling conditions, inclusion criteria, sample size, day of the cycle when biopsies were obtained and statistical analysis applied to the results, among other factors. In sum, all the studies aiming to identify the physiological transcriptomic profile across the menstrual cycle reached the same conclusion: it is possible to accurately catalogue endometria at different stages based on their transcriptomic signatures, specifically the identification of the WOI (see above: ‘A decade of basic research leading to the transcriptomic characterisation of the human endometrium’). Further, the machine-learning predictors used to relate these gene signatures with clinical diagnosis have differed. As an example, in our test, the core of the receptivity diagnosis is powered by 134 ERA genes, while the remaining genes target putative WOI displacements.

Since the publication of our seminal paper identifying the transcriptomic signature of endometrial receptivity (Díaz-Gimeno *et al.*, 2011), six different companies have launched commercial endometrial transcriptomic tests under different acronyms with different evidence. WinTest from INSERM (www.inserm.fr/en) is based on 11 genes detected using RT-qPCR, with four publications demonstrating transcriptomic and clinical consistency (Haouzi *et al.*, 2009; Haouzi, 2015; Bissonnette *et al.*, 2016; Haouzi *et al.*, 2021). ERPeak from Cooper Surgical (USA) (https://fertility.coopersurgical.com/genomics/erpeak-endometrial-receptivity-test/) and ERMap from IGLS (Spain) (https://www.igls.net/es/services/mapa-de-receptividad-endometrial/) both use 40 genes with RT-qPCR supported by the same paper (Enciso *et al.*, 2018). ERT based on 100 genes is commercially available from Yikon (China) (www.yikongenomics.com) but has not been reported in a peer-reviewed publication. BeREADY from Competence Centre on Health Technologies Ltd (Estonia) (https://beready.ccht.ee/) is based on 67 genes supported by one publication in collaboration with our group (Altmäe *et al.*, 2017). BioER from Bioarray (Spain) (https://bioarray.es/es/info/BioEr-TEST-DE-RECEPTIVIDAD-ENDOMETRIAL-60) is based on 72 genes but has not been supported by a peer-reviewed report or proof-of-concept study.

Transcriptomic signature differences have also been considered for endometrial pathologies. In Garcia-Velasco *et al.* (2015), we assessed the endometrial receptivity gene signature in patients with different stages of endometriosis using the ERA test. We concluded that the WOI gene signature does not vary significantly for patients with endometriosis, even considering different stages, compared to controls. Our study also indicated that expression of the gene set was not modified by the presence or stage of endometriosis, but instead by the day of the cycle when the biopsy was obtained. In contradiction to statements by the opponent, this is not a new finding since our group and others have consistently demonstrated that endometrial receptivity is not detrimental to embryo implantation in oocyte recipients with endometriosis, who have outcomes comparable to oocyte recipients without endometriosis (Diaz *et al.*, 2000). Different candidate endometrial markers for endometriosis have been suggested, but whether this is causal or merely consequent of endometriosis, or even whether this has any clinically relevant impact on human embryo implantation, has not been elucidated. Furthermore, oocytes from donors with endometriosis yield poorer PRs than those from donors without endometriosis when donated to otherwise healthy infertile women (Simón *et al.*, 1994), suggesting an embryonic factor is involved in poor prognosis of endometriosis patients.

## Array versus sequencing

Technology is rapidly evolving, and critics should update their knowledge at the same pace. Microarray and PCR-based clinical tests are being replaced by NGS technology (Lowe *et al.*, 2017). In January 2017, the ERA test was moved from microarray-based to NGS-based technology (Clemente-Ciscar *et al.*, 2018), as noted in subsequent diagnostic reports. Results of ERA in the RCT that began in October 2013 and ended in November 2017 were reconfirmed by NGS technology (Simón *et al.*, 2020). Thus, transitioning to new platforms as technology advances is a viable option.

## Method and timing of biopsy and endometrial correction

An important point noted by the opponent is that bulk tissue analysis obtained from a “blind” endometrial biopsy may not be accurate enough to perform the ERA test. Instead, the author offers some guidance by quoting a computational deconvolution system that we co-developed (Suhorutshenko *et al.*, 2018) but is now outdated. The best possible technology currently available to challenge the ERA test in bulk endometrial tissue in any part of the uterine cavity is single-cell RNA sequencing (scRNA-seq). scRNA-seq can promote understanding of how an organ or tissue is arranged at the single-cell level by blending biology and genetics with mathematics, new computational tools and pragmatism. Cells are isolated using microfluidic circuits and nanodroplets, and the mRNA of every cell is sequenced separately. The spatial distribution of RNA or translated proteins can then also be mapped within a tissue or organ (https://data.humancellatlas.org). This technology was chosen as the 2018 breakthrough of the year by *Science*, and its application in the human endometrium is no exception (Wang *et al.*, 2020).

**Figure 2.**
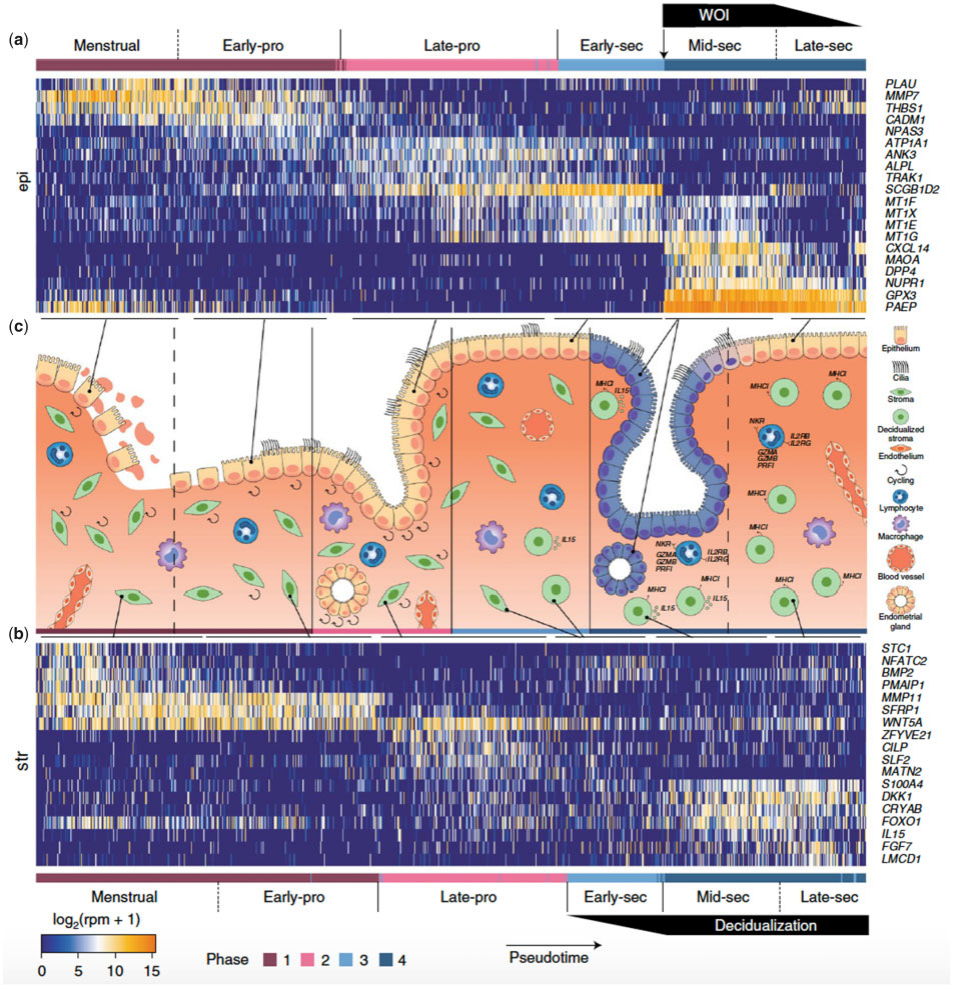
**Temporal transcriptome dynamics of endometrial transformation across the human menstrual cycle by single-cell RNA sequencing (scRNA-seq).** The human WOI opens with abrupt and discontinuous transcriptomic activation in the epithelia. Cells (columns) were ordered by pseudotime. Dashed lines: continuous transition. Solid lines: boundaries between four major phases. Reprinted from Wang et al., 2020 with permissions from Springer Nature. Copyright © 2020, The Author(s), under exclusive licence to Springer Nature America, Inc. Please note that subsequent re-use of this figure is not permitted under this article's Open Access licence. Permission for re-use must be requested from Springer Nature.

In 2020, we reported the characterisation of the human endometrial transcriptome at a single-cell level, revealing cell-specific expression signatures across the menstrual cycle. From 29 healthy oocyte donors, we obtained and analysed 73 180 individual endometrial cells using microfluidics (Fluidigm) or nanodroplets (10× Genomics) (Wang *et al.*, 2020). Employing canonical markers and highly differentially expressed genes, we identified six endometrial cell types: epithelial and endothelial cells, stromal fibroblasts, macrophages, lymphocytes and a novel ciliated epithelial cell type. Further, the signatures revealed that the human WOI involves transcriptomic activation in the epithelia that is both abrupt and discontinuous (Figure 2) (Wang *et al.*, 2020). These cellular-resolution findings confirmed our previous identification from bulk tissue of a unique endometrial receptivity transcriptomic signature (Díaz-Gimeno *et al.* 2011).

The timing of biopsy in relationship to the WOI is also questioned. First, in its development, ERA was compared to the previous gold standard histological methods (n = 128) and concordance against LH peak was superior to histology rating (Diaz-Gimeno *et al.*, 2013) (see above: ‘The endometrial receptivity analysis’). Second, we recommend that endometrial biopsies be obtained at LH + 7 or human chorionic gonadotropin (hCG)+7 in natural cycles or at P + 5 (120 hours) in HRT cycles. This timing maximises the potential to find a receptive WOI, as occurs in 70% of patients analysed at this timing (see above: A decade of ERA clinical application”). Notably, however, the prediction of receptive status within the range of 4 days around the WOI is a major achievement of the ERA test, particularly identifying WOI displacements to guide pET. Some clinics and doctors have performed biopsies earlier or later, and the percentage of receptive cases decreases but the prediction of the WOI is feasible. Importantly, a confirmatory biopsy is not necessary because our algorithm can predict receptivity timing with high accuracy except in specific displacements (<10% of cases analysed). The consistency of the WOI prediction was challenged blindly in one patient through four different biopsies over four months (Cho *et al.*, 2018). After receiving the report for the first biopsy with explicit instructions on how to proceed, the authors instead embarked on a series of additional endometrial biopsies at various timings blinded to us, in opposition to the original recommendation. Biopsies two, three and four all corroborated our initial finding (Stankewicz *et al.*, 2018).

More important than the timing of biopsy is to ensure that endogenous P levels are < 1 ng/mL within 24 hours before the administration of exogenous P in HRT cycles or at the day of hCG administration or LH peak in natural cycles. This step is done to avoid premature activation of the P receptor, which will trigger the initiation of the endometrial receptivity program. Our suggested standard endometrial preparation is HRT because this approach is consistent and reproducible. After menstruation, ovarian quiescence is confirmed by vaginal ultrasound evaluation and E_2_ administration starting from the first or the second day (in Europe, typically E_2_ valerate at a dose of 6 mg/day or E_2_ hemihydrate patches delivering 150 µg every 48 hours; in the United States, oral estrace 200 mg three times daily; there are other possibilities depending on the geographical availability of drugs). Sonographic evaluation and P assessment should be performed 7–10 days after the initiation of endometrial E_2_ preparation. When a ≥ 6-mm trilaminar endometrium is observed with an endogenous P serum level < 1 ng/mL, exogenous P is administered at a dosage and route used by physician/clinic for a period of 5 days (P + 5 or 120 hours). Then, the endometrial biopsy for the ERA test should be obtained. In Europe, typically we use vaginal micronised progesterone (or similar) at a dose of 400 mg/12 h; in the United States, 50 mg intramuscular progesterone daily (or similar) is used. The pET should always be performed using the same protocol as that used for the cycle in which the WOI was diagnosed by the ERA test.

## Progesterone effect

ERA has never been presented independently of progesterone levels (see previous section and Simón *et al.*, 2020). Furthermore, while the route of progesterone administration as well as the serum and tissue P levels are debatable, the activation of the progesterone receptor (PR) is not. PR (A and B) activation is the main driver of the molecular changes that determine the WOI and the initiation of pregnancy. In a collaborative study (von Grothusen *et al.*, 2018), we challenged the ERA prediction ability by blocking the action of P at the cellular level through the antiprogestogen mifepristone, which binds to PR. Mifepristone is approved in many countries for emergency contraception and early first-trimester medical abortion. Indeed, a single dose of 200 mg mifepristone in the immediate postovulatory phase is sufficient to prevent pregnancy by rendering the endometrium refractory or non-receptive without interrupting the normal menstrual cycle (Gemzell-Danielsson *et al.*, 1993, 1994). We demonstrated that a single dose of mifepristone on Day 2 after the LH peak (LH + 2) completely ablates the receptive transcriptomic profile as assessed by the ERA test. Control samples were all staged around receptive stage as would be clinically expected for LH + 7. Treatment samples were all categorised as non-receptive (von Grothusen *et al.*, 2018). Bioinformatic pathway analysis yielded 60 differentially expressed genes within the ERA signature, responsible for the inactivation of the PR and glucocorticoid receptor, consistent with mifepristone action. This finding further demonstrates the capacity of the ERA to identify pharmacologically induced non-receptive endometrium through the blockade of PR (von Grothusen *et al.*, 2018).

## What percentage of women might need the ERA test?

Notably, the opponent quotes an independent study (Mahajan, 2015) as our own study to suggest that we contradict ourselves. He further supports his argument with complex statistical perspectives to make the point that RIF of endometrial origin is so rare that it should not even be treated; his recommendation is to keep trying all over again, pretending to obtain different results.

Regardless of the opponent’s opinion, RIF of endometrial origin is recognised as a concern by all clinicians who transfer euploid embryos that ultimately fail to achieve pregnancy. The ERA test was initially created to solve the problem of our most difficult patients, namely RIF of endometrial origin (see above: ‘A decade of ERA clinical application’) that is estimated to be present in 10% of all IVF cycles (Bellver and Simón, 2018). The RCT exploring, at the first appointment, the cost-effectiveness of this approach compared to FET or fresh ET has been published. Per protocol analysis demonstrated that pET increases the IR at the first embryo transfer by 14.1% (pp) versus FET (*P* = 0.03) and by 18.7% versus fresh ET (*P* = 0.004). LB rates, while not statistically significant, were increased by 13.8% versus FET and 10.5% versus fresh ET (Simón *et al.*, 2020). Thus, it is up to readers to consider if this approach is reasonable to use in all patients.

## A recent multicentre RCT

Crucially, the opponent disproves of our recent RCT because the trial was planned for patients ≤ 37 years old at their first IVF cycle. He argues that such patients are not in need of any additional diagnostic effort to improve clinical results, beyond iterative treatments. We leave it to readers to decide whether there is any room for improvement that will be welcome in this group of patients.

## Effect of embryo cryopreservation

On the cryopreservation of embryos, we strongly disagree with the opponent. Embryo cryopreservation is a consolidated technology that was initially created to store supernumerary embryos, but ultimately changed IVF clinical practice worldwide. Many clinics are now free of ovarian hyperstimulation syndrome thanks to oocyte/embryo cryopreservation (Devroey *et al.*, 2011; Griesinger *et al.*, 2011); as well as fertility preservation is possible in young women (Donnez and Dolmans, 2013); and donor oocytes after storage closed system appear to produced normal obstetric and neonatal outcomes (De Munck *et al.*, 2016). A large multicentre randomised trial assessed obstetrical and perinatal complications, congenital anomaly and neonatal death outcomes following transfer of either fresh or cryopreserved embryos among 2157 women undergoing their first IVF cycle. These outcomes did not differ significantly between groups (Table I) (Shi *et al.*, 2018).

**Table I:** The incidence of obstetrical and perinatal complications, congenital anomaly and neonatal death in fresh embryo transfer compared to frozen embryo transfer groups. Reproduced with permission from Shi *et al.*, *NEJM*, 2018.

Event	Frozen-Embryo Group (N = 1077)	Fresh-Embryo Group (N = 1080)	Absolute Difference (95% CI)	Rate Ratio for Frozen- vs. Fresh- Embryo Transfer (95% CI)	P Value
	no./total no. (%)				
Moderate or severe ovarian hyperstimulation syndrome before biochemical pregnancy	7/1077 (0.6)	22/1080 (2.0)	−1.4 (−2.4 to −0.4)	0.32 (0.14 to 0.74)	0.005
Ectopic pregnancy among biochemical pregnancies	18/671 (2.7)	12/696 (1.7)	1.0 (−0.6 to 2.5)	1.56 (0.76 to 3.21)	0.23
Therapeutic abortion or fetal reduction due to fetal congenital anomalies at 12 to 28 wk of gestation among clinical pregnancies	3/586 (0.5)	4/615 (0.7)	−0.2 (−1.0 to 0.7)	0.79 (0.18 to 3.50)	1.00
Gestational diabetes among clinical pregnancies	18/586 (3.1)	24/615 (3.9)	−0.8 (−2.9 to 1.2)	0.79 (0.43 to 1.44)	0.43
Preeclampsia among clinical pregnancies	26/586 (0.9)	20/615 (3.3)	1.1 (−1.0 to 3.4)	1.36 (0.77 to 2.42)	0.28
Gestational hypertension among clinical pregnancies	5/586 (0.9)	7/615 (1.1)	−0.2 (−1.4 to 0.8)	0.75 (0.24 to 2.35)	0.62
Preterm delivery among clinical pregnancies	91/586 (15.5)	80/615 (13.0)	2.5 (−1.4 to 6.5)	1.19 (0.90 to 1.58)	0.21
Congenital anomalies among live newborns	16/714 (2.2)	26/719 (3.6)	−1.4 (−3.1 to 0.4)	0.62 (0.34 to 1.15)	0.12
Neonatal death among live newborns^a^	2/714 (0.3)	4/719 (0.6)	−0.3 (−0.9 to 0.4)	0.50 (0.09 to 2.74)	0.69

^a^ Neonatal death was defined as the death of a newborn within 28 days after delivery.

The fact is that out of 306 197 ART cycles performed at 456 reporting clinics in the United States in 2018, resulting in 81 478 live-born infants, 103 078 were oocyte- or embryo-cryopreservation cycles in which all resulting oocytes or embryos were frozen for future use (Center for Disease Control and Prevention 2018 Fertility Clinic Success Rates Report). The same trend is observed worldwide except in countries where legislation prevents it, such as UAE. Therefore, arguing a lack of safety of embryo cryopreservation or the use of HRT to justify not investigating the endometrial factor with the ERA test does not stand in 2021.

## Summary and discussion

As physicians, we cannot sit back and ignore the consequences of accepting that failures occur more often than not. This attitude passes a message to our patients that the only way forward is to persevere with doing the same failed approach while expecting a different result. The opponent lives in a unique country in which a patient can, without financial burden, try as many attempts as she (or her doctor) needs, but this is not common throughout the rest of the world. In reality, after the first IVF failure, half of all patients will change doctors. Additionally, the majority of patients in the United States whose health insurance coverage would support a second IVF cycle do not seek further care after a failed treatment (Domar *et al.*, 2018), and in countries where government sponsorship supports multiple IVF cycles, one failed cycle leads a third of patients to discontinue treatment (Brandes *et al.*, 2009). Discontinuation is also three times more likely among patients without IVF insurance coverage than those with IVF insurance coverage (Bedrick *et al.*, 2019). In developing nations, a lack of access to financial support requires patients to self-pay for IVF treatment, which most often means investing their lifetime financial savings in a single treatment. These phenomena underscore the need to improve outcomes of the first IVF attempt.

The notion of ‘add-on’ was created to disprove any attempt to improve the status quo. This concept pretends to ignore that our routine basal IVF results are poor and expensive. The next step has been to group all of them in the same category regardless of their scientific evidence and/or clinical results. Every attempt to improve the status quo from unproven strategies such as praying, scratching or immunological treatment, to others with supportive RCTs such as embryoscope, PGT-A or ERA are considered all the same. The ultimate concern is the economic burden that imposes additional technological efforts to improve our results at the first attempt, obviating the economic pitfall implied in repeating the same process all over again and expecting different results. Yet, add-on treatments should not be implemented without evidence for their benefit. Instead, it is crucial to consider and leverage all existing evidence that may enable the first IVF treatment to be the best possible attempt: after all, it may be their only chance. This approach also circumvents economic concerns, by providing the best possible care from the start, rather than requiring a patient to undergo several costly failed cycles first. Any new evidence-based procedure that offers a ≥ 10% increase in LBR with respect to routine IVF for ≤10% of the cost of a round of IVF should be seriously considered and/or discussed with the patient.

As with previous controversies in medical science, from heart transplants to test-tube babies, attitudes have changed dramatically with time. Progress is historically achieved by the eternal battle between ‘the guardians of faith’ who wish to maintain the status quo, remaining skeptical to any new medical advances even when there is ample room for improvement, and the ‘visionaries’ who see new angles to address the lack of progress in a given field as an opportunity to improve the status quo. Progress is inevitable sooner rather than later.

## Data availability

No new data were generated or analysed in support of this research. The data collected for this manuscript is available in the original papers referenced.

## Authors' roles

M.R-A., D.V. and C.S. contributed to the conception and design of the study. M.R-A., D.V., C.G., J.C. and C.S. contributed to the acquisition of data, drafting of the article and critical review of the final draft. All the authors have approved the final version to be published.

## Funding

The authors declare no funding was given to this work.

## Conflict of interest

M.R., D.V., C.G. and J.C. are employees of Igenomix S.L. C.S. is co-inventor of the patent for gene expression profile (ERA) issued to Igenomix and Head of the Scientific Advisory Board of Igenomix.
